# The effect of parental drinking on alcohol use in young adults: the mediating role of parental monitoring and peer deviance

**DOI:** 10.1111/add.14280

**Published:** 2018-06-27

**Authors:** Liam Mahedy, Georgina J. MacArthur, Gemma Hammerton, Alexis C. Edwards, Kenneth S. Kendler, John Macleod, Matthew Hickman, Simon C. Moore, Jon Heron

**Affiliations:** ^1^ Population Health Sciences, Bristol Medical School University of Bristol United Kingdom; ^2^ Department of Psychiatry and School of Medicine Virginia Institute for Psychiatric and Behavioral Genetics, Virginia Commonwealth University Richmond Virginia United States of America; ^3^ School of Dentistry, College of Biomedical and Life Science Cardiff University United Kingdom

**Keywords:** Alcohol, ALSPAC, parental monitoring, parental transmission, peer deviance, prospective, teenagers

## Abstract

**Background and Aims:**

Evidence demonstrating an association between parental alcohol use and offspring alcohol use from robust prospective studies is lacking. We tested the direct and indirect associations between parental and young adult alcohol use via early alcohol initiation, parental monitoring and associating with deviant peers.

**Design:**

Prospective birth cohort study. Path analysis was used to assess the possible association between parental alcohol use (assessed at 12 years) and alcohol use in young adults (assessed at 18 years) via potential mediators (assessed at 14 and 15.5 years, respectively).

**Setting:**

South West England.

**Participants:**

Data were available on 3785 adolescents and their parents from the Avon Longitudinal Study of Parents and Children.

**Measurements:**

The continuous Alcohol Use Disorders Identification Test (AUDIT) score was used as the primary outcome measure. Maternal alcohol use was defined as light (< 4 units on any day), moderate (≥ 4 units on 1–3 days) and high‐risk (≥ 4 units on ≥ 4 days in 1 week). Partner alcohol use was also defined as light, moderate and high risk. Socio‐economic variables were included as covariates.

**Findings:**

There was strong evidence of a total effect from maternal alcohol use to young adult alcohol use [moderate: *b* = 1.07, 95% confidence interval (CI) = 0.64, 1.49, *P* < 0.001; high risk: *b* = 1.71, 95% CI = 1.07, 2.35, *P* < 0.001]. The majority of this association was explained through early alcohol initiation (moderate: *b* = 0.14, 95% CI = 0.04, 0.25, *P* = 0.01; high risk: *b* = 0.24, 95% CI = 0.07, 0.40, *P* < 0.01) and early alcohol initiation/associating with deviant peers (moderate: *b* = 0.06, 95% CI = 0.02, 0.10, *P <* 0.01; high risk: *b* = 0.10, 95% CI = 0.03, 0.16, *P* < 0.01). There was strong evidence of a remaining direct effect (moderate: *b* = 0.81, 95% CI = 0.39, 1.22, *P* < 0.001; high risk: *b* = 1.28, 95% CI = 0.65, 1.91, *P* < 0.001). A similar pattern of results was evident for partner alcohol use.

**Conclusions:**

Young adults whose parents have moderate or high‐risk alcohol consumption are more likely to consume alcohol than those with parents with lower alcohol consumption. This association appears to be partly accounted for by earlier alcohol use initiation and higher prevalence of association with deviant peers.

## Introduction

Family influences and parental behaviour are critical in shaping how young people use alcohol and whether they experience alcohol‐related negative consequences 1, 2, 3. Notably, parental alcohol use, the provision of alcohol to adolescents, low levels of parental monitoring, a low‐quality parent–child relationship and lack of parental support are all implicated in young people initiating alcohol use earlier and whether they use alcohol during adolescence 1, 4.

While a considerable body of evidence demonstrates the impact of parental alcohol use on child alcohol use, it remains important to gain a greater understanding of the mechanisms underlying the range of potential parental influences, which can influence intervention design and policy 3. However, the impact of parenting on childhood outcomes is limited by imprecise measures and their inconsistent use 1. A recent systematic review of prospective cohort studies 3 identified a deficit of studies that have some capacity for causal inference on the relationship between parental and adolescent alcohol use. Four studies that did have some ‘capacity for causal inference’ (i.e. addressed some, but not all, the following criteria: theory‐driven approach and analysis, analytical rigour and identification and control of sources of bias) reported that parental alcohol use predicts alcohol use in their children and the prevalence of related problems 5, 6, 7, 8. However, the limited use of theory‐driven analysis, small data sets and lack of control for confounding factors mean that there remains considerable uncertainty around the strength of causal inference and the mechanisms of parental influence, or pathways of effect. Moreover, inconsistency was identified around the influence of maternal versus paternal drinking practices 3, 7, 9, 10, 11, 12, thus how aspects of the parent–child relationship influence outcomes remains unclear.

This current prospective cohort study examined the influence of parental alcohol use (recorded when their children were 12 years of age) on characteristics of alcohol use when their children were young adults (18 years of age), and assessed the extent that any associations were mediated by the extent that parents monitor their children's activities, whether children had already initiated alcohol use by 14 years of age and whether children associated with deviant peers at age 15.5 years. The aims were to: (1) estimate the association between parental alcohol use, using separate graded measures of maternal and partner alcohol use and young adult alcohol use and (2) test whether this association was mediated by parental monitoring, early alcohol initiation and associating with deviant peers. We expected to find that (i) parental alcohol use would be associated positively with young adult alcohol use and that (ii) this association would be explained partly through mediators: early alcohol initiation, low parental monitoring and associating with deviant peers.

## Method

### Design

Our approach was informed by recommendations made by Rossow and colleagues 3 and their criteria for strengthening capacity for causal inference. Thus, we have utilized: (1) a theory‐driven analytic approach (examining mechanisms from parental to young adult alcohol use and the inclusion of important covariates; (2) analytical rigour (using path analysis to examine the suggested mechanisms in rich longitudinal data; and (3) minimizing sources of bias (including separate graded measures of maternal and paternal alcohol use collected at an age which could plausibly influence offspring alcohol use and could assess whether a dose–response relationship exists).

Path models, in a structural equation modelling framework, were used to examine the association between parental and young adult alcohol use and whether these associations were mediated through early alcohol initiation, parental monitoring and associating with deviant peers. Maternal reports of their own and their partner's frequency of alcohol use was assessed when the young person was 12 years of age. The young person provided self‐reported information on early alcohol initiation and perceived parental monitoring, both assessed at age 14 years; associating with deviant peers assessed at age 15.5 years; and alcohol use assessed at age 18 years. The clear temporal ordering of exposure, potential mediating variables and outcome helps to rule out the possibility of reverse causality.

### Participants and procedure

We used data from the Avon Longitudinal Study of Parents and Children (ALSPAC), which recruited 14 541 pregnant mothers who resided in the former Avon Health Authority in the South West of England, and had an estimated date of delivery between 1 April 1991 and 31 December 1992. Of the 13 988 offspring alive at 1 year, a small number of participants withdrew from the study (*n* = 24). The sample was restricted further to singletons or first‐born twins, resulting in a starting sample of 13 775. Detailed information about ALSPAC is available online http://www.bris.ac.uk/alspac and in the cohort profiles 13, 14. A fully searchable data dictionary is available on the study's website (http://www.bris.ac.uk/alspac/researchers/data-access/data-dictionary/). Ethical approval for the study was obtained from the ALSPAC Ethics and Law Committee and the Local Research Ethics Committees. Data collection was by postal questionnaires and regular ‘focus’ clinics. Each study was required to submit a research proposal to be approved by the executive committee before gaining access to the ALSPAC data. The overall aims of the study were included in this proposal.

## Measures

### Exposures: parental alcohol use

Mothers completed a postal questionnaire about their daily alcohol consumption during the past week when their children were aged 12 years. Responses, including beverage type and volume consumed, were converted into UK standard units (8 g alcohol). A three‐category variable was created to capture light (drinking < 4 units on any single day; *n* = 3593/6356 (56.5%), moderate (drinking ≥ 4 units on 1–3 days; *n* = 2210/6356 (34.8%) and high‐risk alcohol use (drinking ≥ 4 units on ≥ 4 days in one‐week; *n* = 553/6356 (8.7%).

Maternal reports of partner's frequency of drinking 4 or more units of alcohol was assessed from the same questionnaire. Again, a three‐category variable was created to capture light (drinking ≥ 4 units on ≤ 5 occasions in 1 month; *n* = 2836/5953 (47.6%), moderate (drinking ≥ 4 units on ≥ 5 occasions in 1 month but less than daily; *n* = 2691/5953 (45.2%) and high‐risk drinking (drinking ≥ 4 units daily; *n* = 426/5953 (7.2%). For all analyses, light parental drinking is taken as the reference group. Focusing on these distinct measures allows us to test whether there is a potential dose–response relationship between parental and young adult alcohol use. Further information on parental alcohol use is provided in the Supporting information.

### Offspring outcomes measures: alcohol problem use at age 18 years

Alcohol use was assessed using the Alcohol Use Disorders Identification Test (AUDIT) 15 during a computer‐based session at a research clinic when respondents were aged approximately 18 years [range 0–40, mean = 17.8 years; standard deviation (SD) = 0.46]. The continuous AUDIT total score is used as the main outcome measure because it reflects the extent of alcohol involvement across a broad continuum of severity. However, examining effects on hazardous alcohol use is also of public health importance, as it is a pattern of alcohol use that increases the risk of harmful consequences for the user and others 15, 16. Results examining hazardous alcohol use are presented in secondary analyses.

### Mediators

#### Early alcohol use

Information on alcohol use in early adolescence was assessed using a computer‐assisted survey completed by the young person at approximately 14 years of age (mean = 13.8 years; SD = 0.21) when they attended a research clinic. Participants completed a question asking them how many times they had consumed a whole drink of alcohol during the past 6 months. As we wanted to capture early alcohol initiation, a cut‐off of consuming a whole drink three or more times during the past 6 months was chosen [present/absent, *n* = 1140/5731 (19.9%)].

#### Parental monitoring

Information on parental monitoring was provided by the young person (completed independently of their parents) at the same clinic using a computerized 12‐item self‐report (see Supporting information for a list of items). Internal consistency was good (α = 0.79). Offspring reports were used as a recent study has found that adolescent reports on their parents monitoring were more accurate then parental reports 17.

#### Association with deviant peers

Deviant activity in the young person's peer group was assessed using self‐reports at age 15.5 years (mean = 15.5 years; SD = 0.35) in a research clinic using a computer‐assisted survey. The items were indexed using a questionnaire from the 17‐item Edinburgh Study of Youth Transitions and Crime 18. Response options were ‘yes/no’, with items summed to create a total score. Internal consistency was good (α = 0.86), with higher scores reflecting greater levels of peer deviance.

#### Assumptions in mediation models

Four assumptions were made with respect to confounding in mediation analyses 19. First, control must be made for exposure–outcome confounding. Secondly, control must be made for mediator–outcome confounding. Thirdly, control must be made for exposure–mediator confounding. Finally, there should be no mediator–outcome confounder that is itself affected by the exposure. In the current analyses, all variables were assumed to confound all paths, and these were all assessed or reported to occur before the assessment of the exposure.

### Background covariates

A range of measures, assessed prior to the exposure, were included as covariates in the model. Measures of socio‐economic position (SEP) were recorded in maternal self‐report questionnaires administered during pregnancy. These included: maternal age at delivery, SEP (unskilled/semiskilled manual; skilled manual/non‐manual; managerial/technical; and professional), maternal education (< O‐level: indicating no formal qualification; O‐level: indicating completion of school examinations at 16 years of age; and > O‐level: indicating completion of college or university education at or after age 18 years), maternal smoking during first trimester in pregnancy (yes/no), housing tenure (mortgaged, subsidized renting and private renting) and household income (ranging in quintiles from the lowest to highest 20%).

### Statistical analysis

**Figure 1:**
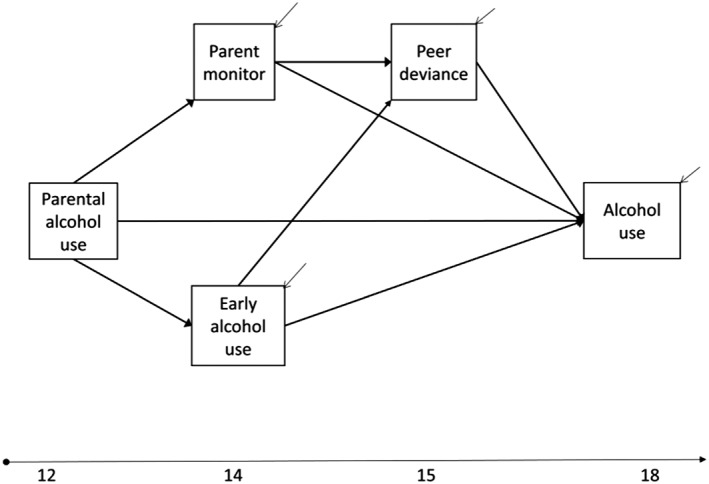
Hypothesized model showing associations among parental alcohol use, parental monitoring, early alcohol initiation, peer deviance and drinking in young adulthood, adjusted for background covariates. Model adjusted for gender, maternal age at delivery, maternal smoking in pregnancy, maternal education, family income, housing tenure, socio‐demographic position (all assessed in pregnancy)

**Figure 2:**
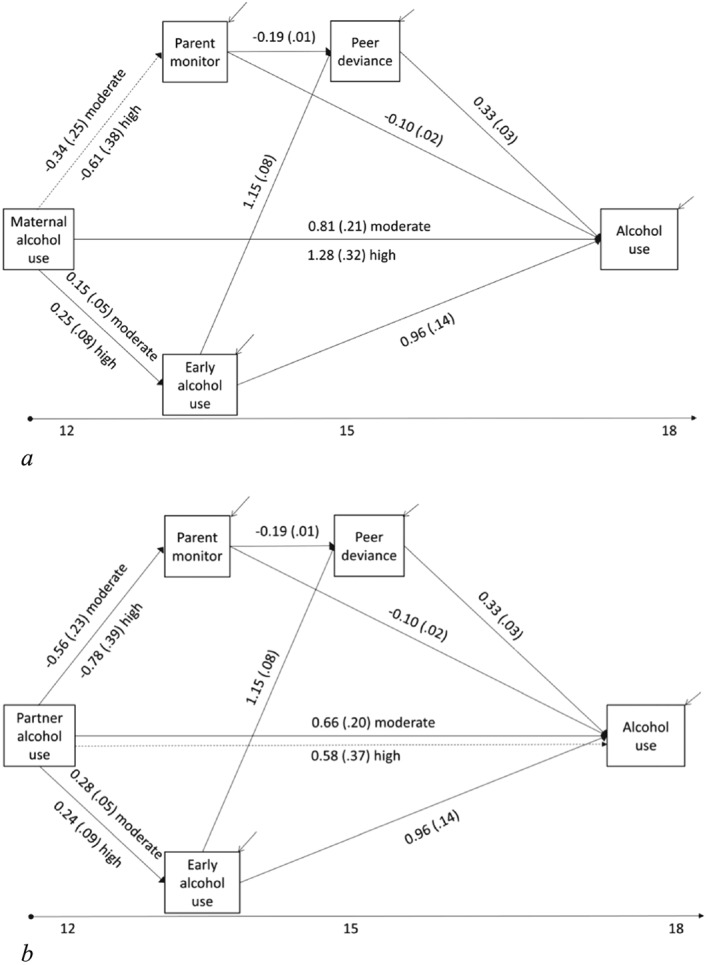
(a) Maternal alcohol use; (b) partner alcohol use. Path model showing the direct and indirect effects of parental alcohol use on young adult alcohol use through parental monitoring, peer deviance and alcohol use early in early adolescence, while adjusting for gender, maternal age at delivery, maternal smoking during pregnancy, family income, socio‐economic position, housing tenure and maternal education (inclusion of background covariates are not shown for ease of interpretation) (n = 3785). Dotted directional arrows indicate insufficient evidence of an association. Direct and indirect effects are obtained using the product of coefficients approach, whereby the indirect effects are derived by multiplying the parameters along each of the paths from exposure to outcome. For example, the indirect effect from partner moderate alcohol use to young adult alcohol use via early alcohol initiation and peer deviance is calculated by multiplying the coefficients along those paths (0.28 × 1.15 × 0.33 = 0.11)

A mediation model (Fig. 1) was run to examine whether there was evidence of an indirect pathway from parental alcohol use, measured prior to adolescence, to alcohol use at age 18. Although alcohol initiation is operationalized as a binary measure, when using the robust weighted least squares (WLSMV) estimator it is treated as a manifestation of an underlying latent continuous response variable. This approach has been conducted using the traditional ‘product of coefficients’ approach to examine mediation models 20. Direct and indirect effects can be obtained. Indirect effects are derived by multiplying the parameters along each of the paths from exposure to outcome (example presented in Fig. 2 footnote). Unstandardized linear regression coefficients are reported for the total, total indirect, specific indirect and direct effects. Direct and indirect effects reflect a change in the continuous AUDIT score. Analyses were conducted in Mplus version 8 21.

### Missing data

Data were available on 4376 participants before adjusting for covariates (all assessed in the antenatal period). The inclusion of covariates had a minimal impact on the fully adjusted sample size of 3785 (2124 females and 1661 males). Supporting information, Figure S1 shows a flow‐chart of data retention in ALSPAC. The missing at random (MAR) assumption was made more plausible by the inclusion of socio‐demographic variables in the fully adjusted models. Missing data were handled using the expectation maximization algorithm based on the assumption of being ‘missing at random’ on exogenous covariates 22. The pairwise present method uses polychoric correlations for pairwise present data and ignores only the missing values involved in the two variables, as opposed to all the information concerning the case 22.

Models were run using inverse probability weighting (IPW) 23 to address any potential bias caused by participant dropout. Weights were derived from a logistic regression model between a set of measures assessed in pregnancy that were independently predictive of missing data and/or variables in the analysis (maternal severe depression, maternal smoking in pregnancy, parity, use of car, marital status, damp/mould/condensation on internal walls of accommodation) and participants who were in the analysis (*n* = 3785/9556). Additional information on the IPW analyses are presented in the Supporting information. As it is not possible to incorporate bias‐corrected bootstrapped confidence intervals and weights into the same model, models using IPW are reported as the main results. Sensitivity analyses using bias‐corrected bootstrapped confidence intervals are presented in Supporting information, Table S1. As a further sensitivity check, we investigated the effect of the missing data technique on the complete case sample (Supporting information, Table S2).

### Secondary analyses

Hazardous alcohol use in young adulthood was examined using the standard cut‐off of 8 or more for ‘hazardous’ alcohol use 15.

## Results

### Descriptive data

Table 1 shows evidence of a relationship between socio‐demographic variables and missing data on alcohol use at age 18 years. Overall, participants included in the study were more likely to be female, later alcohol initiation and less association with deviant peers. Participants were also more likely to be from higher‐income families, higher social class, maternal partner consuming lower levels of alcohol and living in mortgaged accommodation. Their mothers had lower levels of education, were less likely to smoke in pregnancy and were older at age of delivery.

**Table 1:** Descriptive characteristics for the complete sample and partial responders [based on the sample size used in the inverse probability weighting (IPW), n = 9556].

	Available (n = 3785)	Not available (n = 5771)	
Categorical measures	n (%)	n (%)	Statistical test
Maternal alcohol use			
Yes	1049 (34.7)	916 (35.4)	χ^2^_(1)_ = 0.32
Partner alcohol use			
Yes	1249 (43.6)	1129 (47.4)	χ^2^_(1)_ = 7.57**
Early alcohol initiation			
Yes	605 (18.1)	467 (21.8)	χ^2^_(1)_ = 10.95**
Gender			
Males	1723 (43.8)	3039 (54.0)	χ^2^_(1)_ = 94.6***
Income			
Lowest 20%	465 (13.3)	909 (22.2)	
2	639 (18.2)	844 (20.6)	
3	740 (21.1)	769 (18.8)	
4	800 (22.8)	794 (19.4)	
Highest 20%	861 (24.6)	780 (19.0)	χ^2^_(4)_ = 131.2***
Social			
Unskilled or semi‐skilled	136 (3.7)	294 (6.4)	
Skilled manual or non‐manual	1148 (31.4)	1879 (40.6)	
Managerial and technical	1681 (45.9)	1926 (41.7)	
Professional	694 (19.0)	524 (11.3)	χ^2^_(3)_ = 165.0***
Housing tenure			
Mortgaged	3264 (85.5)	3766 (71.7)	
Subsidized rent	276 (7.2)	573 (10.9)	
Private rent	277 (7.3)	914 (17.4)	χ^2^_(2)_ = 259.6***
Maternal education			
< O‐level	1820 (47.8)	1656 (33.0)	
O‐level	1295 (34.0)	1761 (35.1)	
> O‐level	692 (18.2)	1603 (31.9)	χ^2^_(2)_ = 279.0***
Smoking in pregnancy			
Yes	746 (19.0)	1802 (32.0)	χ^2^_(1)_ = 200.42***
*Continuous measures*	*Mean (SD)*	*Mean (SD)*	*Mean difference*
Peer deviance			
	3.65 (3.41)	4.34 (3.88)	0.69 (0.48, 0.89)
Parental monitoring			
	34.09 (6.22)	32.82 (6.65)	1.27 (0.93, 1.60)
Maternal age			
	29.35 (4.6)	27.77 (4.86)	1.57 (1.38, 1.77)

** *P* < 0.01;

*** *P* < 0.001.

SD = standard deviation.

### Path models

Path coefficients demonstrating the association between parental alcohol use and young adult alcohol use via the hypothesized mediators are presented in Fig. 2.

Table 2 shows robust evidence of a total indirect effect from maternal alcohol use to young adult alcohol use through parental monitoring, early alcohol use and peer deviance (overall indirect effect from maternal moderate alcohol use: *b* = 0.26, 95% CI = 0.08, 0.44, *P* = 0.001; and maternal high‐risk alcohol use: *b* = 0.43, 95% CI = 0.15, 0.71, *P* < 0.01). When examining the specific indirect effects for maternal alcohol use, the majority of the indirect effect was accounted for through early alcohol use (moderate alcohol use: *b* = 0.14, 95% CI = 0.04, 0.25, *P* = 0.01; high‐risk alcohol use: *b* = 0.24, 95% CI = 0.07, 0.40, *P* < 0.01) and early alcohol use and later peer deviance (moderate alcohol use: *b* = 0.06, 95% CI = 0.02, 0.10, *P* < 0.01; high‐risk alcohol use: *b* = 0.10, 95% CI = 0.03, 0.16, *P* < 0.01). There was further evidence of a remaining direct effect (moderate alcohol use: *b* = 0.81, 95% CI = 0.39, 1.22, *P* < 0.001; high‐risk alcohol use: *b* = 10.28, 95% CI = 0.65, 1.91, *P* < 0.001).

**Table 2:** Total, direct and indirect effects of parental alcohol use on young adult alcohol use (n = 3785).

	Maternal alcohol use			Partner alcohol use				
	Moderate‐risk		High‐risk		Moderate‐risk		High‐risk	
	b (95% CI)	P	b (95% CI)	P	b (95% CI)	P	b (95% CI)	P
Total effect	1.07 (0.64, 1.49)	< 0.001	1.71 (1.07, 2.35)	< 0.001	1.12 (0.72, 1.51)	< 0.001	1.03 (0.31, 1.74)	< 0.001
Total indirect effect	0.26 (0.08, 0.44)	0.001	0.43 (0.15, 0.71)	< 0.01	0.46 (0.28, 0.64)	< 0.001	0.44 (0.15, 0.74)	< 0.01
Specific indirect effects								
Parental monitoring	0.03 (−0.02, 0.08)	0.18	0.06 (−0.02, 0.13)	0.12	0.06 (0.01, 0.10)	0.02	0.08 (0.00, 0.16)	0.06
Early alcohol initiation	0.14 (0.04, 0.25)	00.01	0.24 (0.07, 0.40)	< 0.01	0.26 (0.15, 0.38)	< 0.001	0.23 (0.05, 0.41)	0.01
Parental monitoring–peer deviance	0.02 (−0.01, 0.05)	0.17	0.04 (−0.01, 0.09)	0.11	0.04 (0.01, 0.06)	0.02	0.05 (0.00, 0.10)	0.05
Early alcohol use–peer deviance	0.06 (0.02, 0.10)	< 0.01	0.10 (0.03, 0.16)	< 0.01	0.11 (0.06, 0.15)	< 0.001	0.09 (0.02, 0.16)	0.01
Direct effect	0.81 (0.39, 1.22)	< 0.001	1.28 (0.65, 1.91)	< 0.001	0.66 (0.26, 1.05)	< 0.001	0.58 (−0.15, 1.31)	0.12

Unstandardized coefficients and 95% confidence intervals are presented; models adjusted for gender, maternal age at delivery, maternal smoking in pregnancy, maternal education, socio‐economic position, housing tenure and family income. CI = confidence interval.

We found a similar pattern of findings when examining partner alcohol use. There was weak evidence to suggest that parental monitoring accounted for some of the association between partner alcohol use and young adult alcohol use (moderate alcohol use: *b* = 0.06, 95% CI = 0.01, 0.10, *P* = 0.02; high‐risk alcohol use: *b* = 0.08, 95% CI = 0.00, 0.16, *P* = 0.06) and through parental monitoring and later peer deviance (moderate alcohol use: *b* = 0.04, 95% CI = 0.01, 0.06, *P* = 0.02; high‐risk alcohol use: *b* = 0.05, 95% CI = 0.00, 0.10, *P* < 0.05). Finally, there was insufficient evidence to suggest a direct effect of partner high‐risk alcohol use and young adult alcohol use (*b* = 0.58, 95% CI = −0.15, 1.31, *P* = 0.12).

### Missing data: sensitivity analyses

Overall, findings from the models using bias‐corrected bootstrapped confidence intervals (Supporting information, Table S1) and the complete case analyses (Supporting information, Table S2) supported the main findings. There was strong evidence of a total indirect effect from parental alcohol use (moderate and high‐risk) to young adult alcohol use through parental monitoring, early alcohol use and peer deviance. There was also evidence of a remaining direct effect apart from partner high‐risk alcohol use and young adult alcohol use.

### Secondary analyses

Overall, the findings focusing upon hazardous alcohol use in young adulthood supported the findings from the main analyses (Supporting information, Table S3). There was strong evidence of a total indirect effect from parental alcohol use (moderate and high‐risk) to young adult alcohol use through parental monitoring, early alcohol use and peer deviance. There was also evidence of a remaining direct effect for all models. Further information is provided in the Supporting information.

## Discussion

We found strong evidence of an indirect effect between parental alcohol use and their children's alcohol use in young adulthood, primarily through early alcohol initiation and associating with deviant peers. Overall, there was evidence also of a direct pathway from parental alcohol use to alcohol use in young adulthood, suggesting an independent effect of parental alcohol use on young adult alcohol use not explained through our hypothesized mediators. There was insufficient evidence to indicate a direct pathway from partner high‐risk alcohol use to alcohol use in young adulthood.

### Comparison with previous studies

Our findings support those of systematic reviews and meta‐analyses evidencing an association between parental alcohol use and both the initiation of alcohol use and levels of alcohol use among adolescent children 3, 4, 24. Building upon social cognitive theory and recent models of cognitive transference 25, 26, 27, low parental monitoring may increase the likelihood of early alcohol initiation and association with deviant peers via the perception of more tolerant or more permissive parental attitudes towards adolescent alcohol use, which may be modelled directly by young people or indirectly through the transference of attitudes that are positive or approving towards alcohol use and its consequences. Indeed, even before initiation of alcohol use, children are exposed to motives and expectancies as to the effects of alcohol which can originate from parental observation and shape later behaviour 27. All these factors, if present, can reduce the risk of initiation of alcohol use and alcohol misuse during adolescence 4; thus, as a corollary, if absent, could increase the likelihood of early initiation of adolescent drinking.

As children transition to adolescence, relationships with peers take on a more important role. A number of studies demonstrate a role for the selection of peers with similar drinking levels 28, 29, 30, 31, and the influences of perceived or actual drinking behaviours of peers 32. A social learning perspective would suggest that alcohol use with deviant alcohol‐using role models can contribute to the maintenance or escalation of use via modelling of behaviour 33 or the perception of peer norms 34. Thus, early initiation and continuation of drinking may encourage the selection of similar higher‐level drinking peers and subsequent influences that further increase alcohol use 35.

Notably, in this study, by assessing separate measures of maternal and paternal alcohol use, we have identified distinct effects of each parent/guardian, with mothers having both a direct and indirect role. The role of maternal partners was less consistent. There was evidence of both indirect and direct effect of partners who were moderate drinkers, while there was evidence of an indirect effect only for partners who were high‐risk drinkers. Overall, approximately 25% of the total effect for models examining maternal alcohol use, and approximately 40% of the total effect for models examining partner alcohol use were explained through the hypothesized mediators.

To date, findings regarding the comparative impact of each parent are somewhat mixed, with studies reporting greater impacts of maternal 8, 10, 11, 36 versus paternal alcohol use and with others reporting associations between paternal, but not maternal, alcohol use and alcohol use in adolescence 7, 12. This may be due, in part, to the use of different measures of alcohol use among adolescents and parents between studies, using maternal self‐reports and maternal reports of partner alcohol use, assessing alcohol use at different time‐points and geographic location of studies, which could influence cultural practices.

### Strengths and limitations

This study has a number of strengths, including the use of: (1) theory to inform the analytical approach; (2) analytical rigour; and (3) minimizing sources of bias. However, we acknowledge several limitations. Although we cannot rule out the possibility that exclusion of parents without complete information might have biased our findings for reported alcohol use, there was minimal change to the models when differential dropout was accounted for using inverse probability weighting. Furthermore, the inclusion of a number of covariates (which have been shown to predict dropout at age 18 years) had minimal impact on the association between parental and young adult alcohol use, indicating that the adjusted models including IPW should be addressing the impact of non‐response bias.

Secondly, self‐reported alcohol use is often under‐reported or exaggerated 37; however, participants completed questionnaires individually and were assured of the anonymity of their responses. Moreover, there is some evidence to suggest that self‐reported alcohol use is a reliable and valid method 38. Thirdly, it was not possible to include interactive or additive effects of parental alcohol use, as reports of parental alcohol use were assessed over different time‐periods (i.e. units per week/month). Although the use of separate self‐reported measures would have been preferable, maternal reports of their partners’ alcohol use was used because of (1) the rate of attrition in partner reporting and (2) the very good inter‐rater agreement between parental reports of partners drinking practices reported previously in ALSPAC 39. Fourthly, the indirect effects were small in magnitude. However, as the ‘causal’ process becomes more distal, the size of the effect typically becomes smaller as it becomes more likely that it is transmitted through additional links in the causal chain, affected by competing causes and by random factors 40, such as parental supply of alcohol and access to alcohol in the home 41.

### Summary and implications

We strengthen the evidence on potential ‘transmission’ of adverse alcohol use from parents to their children. Our study incorporated many of the guidelines outlined by the Rossow *et al*. systematic review, and now represents the strongest evidence of an association between parental and offspring alcohol use to date. Although we have incorporated several guidelines for strengthening causal inference in observational studies, we do not seek to over‐interpret our results as drawing causal inference. We have identified direct and indirect pathways of parental influence on adolescent alcohol use. Our findings indicate a need to focus prevention efforts on parental alcohol use, behaviours and attitudes, as well as the influence of parental alcohol use through early initiation and peer associations and influences. As combined parent and child alcohol prevention programmes have been found to be effective in reducing the initiation and frequency of alcohol use during adolescence 42, 43, targeting of prevention efforts early in the life course, while engaging parents, may play an important role in delaying initiation of alcohol use and reducing susceptibility to peer influences throughout adolescence, thus reducing alcohol‐associated harms over the longer term.

## Declaration of interests

None.

## Supporting information


**Figure S1** Flow‐chart showing available data for parental alcohol use, parental monitoring, early alcohol initiation, associating with deviant peers, young adult alcohol use and all potential confounders.
**Table S1** Total, direct and indirect effects of parental alcohol use on young adult alcohol use (n = 3785).
**Table S2** Total, direct and indirect effects of parental alcohol use on young adult alcohol use (complete case sample, n = 1994).
**Table S3** Total, direct and indirect effects of parental alcohol use on young adult hazardous alcohol use (n = 3785).

## References

[1] Ryan S. M. , Jorm A. F. , Lubman D. I. Parenting factors associated with reduced adolescent alcohol use: a systematic review of longitudinal studies. Aust NZ J Psychiatry 2010; 44: 774–783. 10.1080/00048674.2010.501759 20815663

[2] Grigsby T. J. , Forster M. , Unger J. B. , Sussman S. Predictors of alcohol‐related negative consequences in adolescents: a systematic review of the literature and implications for future research. J Adolesc 2016; 48: 18–35. 26871952 10.1016/j.adolescence.2016.01.006 PMC4779657

[3] Rossow I. , Keating P. , Felix L. , McCambridge J. Does parental drinking influence children's drinking? A systematic review of prospective cohort studies. Addiction 2016; 111: 204–217. 26283063 10.1111/add.13097 PMC4832292

[4] Yap M. B. H. , Cheong T. W. K. , Zaravinos‐Tsakos F. , Lubman D. I. , Jorm A. F. Modifiable parenting factors associated with adolescent alcohol misuse: a systematic review and meta‐analysis of longitudinal studies. Addiction 2017; 112: 1142–1162. 28178373 10.1111/add.13785

[5] Pears K. C. , Capaldi D. M. , Owen L. D. Substance use risk across three generations: the roles of parent discipline practices and inhibitory control. Psychol Addict Behav 2007; 21: 373–386. 17874888 10.1037/0893-164X.21.3.373 PMC1988842

[6] Latendresse S. J. , Rose R. J. , Viken R. J. , Pulkkinen L. , Kaprio J. , Dick D. M. Parenting mechanisms in links between parents’ and adolescents’ alcohol use behaviors. Alcohol Clin Exp Res 2008; 32: 322–330. 18162066 10.1111/j.1530-0277.2007.00583.x PMC2504716

[7] Mares S. H. W. , van der Vorst H. , Engels R. C. M. E. , Lichtwarck‐Aschoff A. Parental alcohol use, alcohol‐related problems, and alcohol‐specific attitudes, alcohol‐specific communication, and adolescent excessive alcohol use and alcohol‐related problems: an indirect path model. Addict Behav 2011; 36: 209–216. 21084165 10.1016/j.addbeh.2010.10.013

[8] Alati R. , Baker P. , Betts K. S. , Connor J. P. , Little K. , Sanson A. *et al* The role of parental alcohol use, parental discipline and antisocial behaviour on adolescent drinking trajectories. Drug Alcohol Depend 2014; 134: 178–184. 24479151 10.1016/j.drugalcdep.2013.09.030

[9] Webster Harburg E. , Gleiberman L. , Schork A. , DiFranceisco W. Familial transmission of alcohol use: I. Parent and adult offspring alcohol use over 17 years—Tecumseh, Michigan. J Stud Alcohol Drugs 1989; 50: 557–566. 10.15288/jsa.1989.50.557 2586109

[10] Casswell S. , Pledger M. , Pratap S. Trajectories of drinking from 18 to 26 years: identification and prediction. Addiction 2002; 97: 1427–1437. 12410783 10.1046/j.1360-0443.2002.00220.x

[11] Poelen E. A. P. , Scholte R. H. J. , Willemsen G. , Boomsma D. I. , Engels R. C. M. E. Drinking by parents, siblings, and friends as predictors of regular alcohol use in adolescents and young adults: a longitudinal twin‐family study. Alcohol Alcohol 2007; 42: 362–369. 17537828 10.1093/alcalc/agm042

[12] Poelen E. A. P. , Engels R. C. M. E. , Scholte R. H. J. , Boomsma D. I. , Willemsen G. Predictors of problem drinking in adolescence and young adulthood. Eur Child Adolesc Psychiatry 2009; 18: 345–352. 19205784 10.1007/s00787-009-0736-x

[13] Boyd A. , Golding J. , Macleod J. , Lawlor D. A. , Fraser A. , Henderson J. *et al* Cohort profile: the ‘Children of the 90s’—the index offspring of the Avon Longitudinal Study of Parents and Children. Int J Epidemiol 2013; 42: 111–127. 22507743 10.1093/ije/dys064 PMC3600618

[14] Fraser A , Macdonald‐Wallis C , Tilling K , Boyd A , Golding J , Davey Smith G . *et al* Cohort profile: the Avon Longitudinal Study of Parents and Children: ALSPAC mothers cohort. Int J Epidemiol 2013;42:97–110. 22507742 10.1093/ije/dys066 PMC3600619

[15] Babor T. , Higgins‐Biddle J. C. , Saunders J. B. , Monteiro M. G. The Alcohol Use Disorders Identification Test: Guidelines for Use in Primary Care. Geneva: World Health Organization; 2001, pp. 1–40.

[16] Reid M. C. , Fiellin D. A. , O’Connor P. G. Hazardous and harmful alcohol consumption in primary care. Arch Intern Med 2011; 159: 1681–1689. 10.1001/archinte.159.15.1681 10448769

[17] Abar C. C. Examining the relationship between parenting types and patterns of student alcohol‐related behavior during the transition to college. Psychol Addict Behav 2012; 26: 20–29. 21842968 10.1037/a0025108 PMC3243795

[18] Smith D. J. , McVie S. Theory and method in the Edinburgh Study of Youth Transitions and Crime. Br J Criminol 2003; 43: 169–195.

[19] VanderWeele T. J. Mediation analysis: a practitioner's guide. Annu Rev Public Health 2016; 37: 17–32. 26653405 10.1146/annurev-publhealth-032315-021402

[20] MacKinnon D. P. Contrasts in Multiple Mediator Models. Multivariate Applications in Substance Use Research: New Methods for New Questions. Mahwah, NJ: Lawrence Erlbaum Associates Publishers; 2000, pp. 141–160.

[21] Muthén L. K. , Muthén B. O. User's Guide, 7th edn. Los Angeles, CA: Muthén & Muthén; 2016.

[22] Asparouhov T. , Muthén BO . Weighted least squares estimation with missing data. MplusTechnical Appendix 2010;1–10.

[23] Seaman S. R. , White I. R. Review of inverse probability weighting for dealing with missing data. Stat Methods Med Res 2013; 22: 278–295. 21220355 10.1177/0962280210395740

[24] Allen M. , Donohue W. A. , Griffin A. , Ryan D. , Turner M. M. M. Comparing the influence of parents and peers on the choice to use drugs. Crim Justice Behav 2003; 30: 163–186.

[25] Bandura A. Social Foundations of Thought and Action: A Social Cognitive Theory. Englewood Cliffs, NJ: Prentice‐Hall, Inc.; 1986.

[26] Campbell J. M. , Oei T. P. A cognitive model for the intergenerational transference of alcohol use behavior. Addict Behav 2010; 35: 73–83. 19783372 10.1016/j.addbeh.2009.09.013

[27] Campbell J. M. , Oei T. P. The intergenerational transference of alcohol use behaviour from parents to offspring: a test of the cognitive model. Addict Behav 2010; 35: 714–716. 20219287 10.1016/j.addbeh.2010.02.001

[28] Wang C. , Hipp J. R. , Butts C. T. , Jose R. , Lakon C. M. Alcohol use among adolescent youth: the role of friendship networks and family factors in multiple school studies. PLOS ONE 2015; 10: e0119965. 25756364 10.1371/journal.pone.0119965 PMC4355410

[29] Mercken L. , Steglich C. , Sinclair P. , Holliday J. , Moore L. A longitudinal social network analysis of peer influence, peer selection, and smoking behavior among adolescents in British schools. Health Psychol 2012; 31: 450–459. 22251218 10.1037/a0026876

[30] Mundt M. P. , Mercken L. , Zakletskaia L. Peer selection and influence effects on adolescent alcohol use: a stochastic actor‐based model. BMC Pediatr 2012; 12: 115. 22867027 10.1186/1471-2431-12-115 PMC3469361

[31] Huang G. C. , Soto D. , Fujimoto K. , Valente T. W. The interplay of friendship networks and social networking sites: longitudinal analysis of selection and influence effects on adolescent smoking and alcohol use. Am J Public Health 2014; 104: e51–9. 10.2105/AJPH.2014.302038 PMC4103209 24922126

[32] Leung R. K. , Toumbourou J. W. , Hemphill S. A. The effect of peer influence and selection processes on adolescent alcohol use: a systematic review of longitudinal studies. Health Psychol Rev 2014; 8: 426–457. 25211209 10.1080/17437199.2011.587961

[33] Petraitis J. , Flay B. , Miller T. Reviewing theories of adolescent substance use: organizing pieces in the puzzle. Psychol Bull 1995; 117: 67–86. 7870864 10.1037/0033-2909.117.1.67

[34] Halim A. , Hasking P. , Allen F. The role of social drinking motives in the relationship between social norms and alcohol consumption. Addict Behav 2012; 37: 1335–1341. 22958866 10.1016/j.addbeh.2012.07.004

[35] Osgood D. W. , Ragan D. T. , Wallace L. , Gest S. D. , Feinberg M. E. , Moody J . Peers and the emergence of alcohol use: influence and selection processes in adolescent friendship networks. J Res Adolesc 2013; 23: 500–512. 10.1111/jora.12059 PMC3844135 24307830

[36] Macleod J. , Hickman M. , Bowen E. , Alati R. , Tilling K. , Smith G. D. Parental drug use, early adversities, later childhood problems and children's use of tobacco and alcohol at age 10: birth cohort study. Addiction 2008; 103: 1731–1743. 18705686 10.1111/j.1360-0443.2008.02301.x

[37] Tourangeau R. , Yan T. Sensitive questions in surveys. Psychol Bull 2007; 133: 859–883. 17723033 10.1037/0033-2909.133.5.859

[38] Del Boca F. K. , Darkes J. The validity of self‐reports of alcohol consumption: state of the science and challenges for research. Addiction 2003; 98: 1–12. 10.1046/j.1359-6357.2003.00586.x 14984237

[39] Mahedy L. , Hammerton G. , Teyhan A. , Edwards A. C. , Kendler K. S. , Moore S. C. *et al* Parental alcohol use and risk of behavioral and emotional problems in offspring. PLOS ONE 2017; 12: 1–15. 10.1371/journal.pone.0178862 PMC5460848 28586358

[40] Shrout P. E. , Bolger N . Mediation in experimental and nonexperimental studies: new procedures and recommendations. Psychol Methods 2002; 7: 422–445. 12530702

[41] Chan G. C. K. , Leung J. , Connor J. , Hall W. , Kelly A. B. Parental supply of alcohol and adolescent drinking: a multilevel analysis of nationally representative data. BMC Public Health 2017; 17: 560. 28599649 10.1186/s12889-017-4472-8 PMC5466780

[42] Koning I. M. , Vollebergh W. A. M. , Smit F. , Verdurmen J. E. E. , Van Den Eijnden R. J. J. M. , Ter Bogt T. F. M. *et al* Preventing heavy alcohol use in adolescents (PAS): cluster randomized trial of a parent and student intervention offered separately and simultaneously. Addiction 2009; 104: 1669–1678. 21265908 10.1111/j.1360-0443.2009.02677.x

[43] Newton N. C. , Champion K. E. , Slade T. , Chapman C. , Stapinski L. , Koning I. *et al* A systematic review of combined student‐ and parent‐based programsto prevent alcohol and other drug use among adolescents. Drug Alcohol Rev 2017; 36: 337–351. 28334456 10.1111/dar.12497

