# Supplementary material for: The effect of parental drinking on alcohol use in young adults: the mediating role of parental monitoring and peer deviance
Source: Addiction. 2018 Jun 27;113(11):2041–50. doi: 10.1111/add.14280 (PMC6176713; doi:10.1111/add.14280)
Supplement: Supplementary file 1 — Figure S1 Flow‐chart showing available data for parental alcohol use, parental monitoring, early alcohol initiation, associating with deviant peers, young adult alcohol use and all potential confounders. Table S1 Total, direct and indirect effects of parental alcohol use on young adult alcohol use (n = 3785). Table S2 Total, direct and indirect effects of parental alcohol use on young adult alcohol use (complete case sample, n = 1994). Table S3 Total, direct and indirect effects of parental alcohol use on young adult hazardous alcohol use (n = 3785). [file ADD-113-2041-s001.docx]

*Parental alcohol use*

Maternal alcohol use: Reponses were converted into units e.g., ½ pint of ‘beer, larger, or cider’, ‘sherry and others’, one pub measure ‘spirit’, and ‘other alcoholic drinks’ reflects 1 unit of alcohol; one glass of wine reflects two units of alcohol, ‘ready mix drinks’ reflects 1.5 units of alcohol; and ‘low alcoholic drinks’ reflects 0.5 of a unit of alcohol. One UK unit of alcohol corresponds to 8 grams of alcohol.

Partner alcohol use: ‘How many days in the past month do you think he had the equivalent of 2 pints of beer, 4 glasses of wine or 4 pub measures of spirit?’ Response options were: *none*, *1-2 days*, *3-4 days*, *5-10 days*, *more than 10 days*, and *every day*

*Parental monitoring items*

1. How often do your carers / parents know what you do during your free time?
2. How often do your carers / parents know what you spend your money on?
3. How often, during the last month, have your carers / parents been unaware of where you were at night?
4. How often do you keep secrets from your carers / parents about what you do during your free time?
5. How often do you keep things from your carers / parents about what you do during nights and weekends?
6. How often do you tell your carers / parents about what you did and where you went during the evening?
7. How often do your carers / parents ask you about what happened during your free time?
8. How often were conversations about your spare time started by your carers / parents during the past month?
9. How often do your carers / parents take the time to listen to you when you talk about what happened during your free time?
10. How often do you have to have your carers / parents’ permission before you go out on weeknights?
11. How often do your carers / parents demand to know where you are in the evenings, who you are going to be with and what you are going to do before you go out?
12. How often do your carers / parents ask you to tell them how you spend your money?

Response options for each question were: ‘never’, ‘hardly ever’, ‘sometimes’, ‘most of the time’, and ‘always’. Items were reverse coded, if necessary, so that higher scores reflected a greater degree of parental monitoring.

Pregnant women enrolled in ALSPAC Phase 1 *N*=14,541

Offspring alive at one year *N*=13,954

Participants invited to clinic (mean age: 17y 10m) *N*=9,556

Sample used to derive weights

Participants attended clinic *N*=4,878

Participants had available alcohol data at age 18 *N*=3,927

Partial data available for exposure, mediators and outcome and complete data available for confounders *N*=3,785

Sample used in main analyses

Complete data on confounders *N*=1,994

*Figure S1. Flowchart showing available data for parental alcohol use, parental monitoring, early alcohol initiation, associating with deviant peers, young adult alcohol use and all potential confounders*

*Inverse probability weighting*

Inverse probability weighting (IPW) was used to investigate the possible influence of selective participation on our estimates of association between parental alcohol use and alcohol use in young adulthood. Estimates were weighted to account for probabilities of nonresponse in the alcohol outcome. The process of weighting allows a greater weight to be given to individuals who have similar prenatal characteristics to those individuals who are likely to subsequently be lost to the study.

Response rates differed according to mother had severe depression, maternal smoking in pregnancy, parity, use of car, marital status, damp/mould/condensation on walls of house making the MAR assumption more plausible. The reciprocal of the predicted probabilities from this model were used as sampling weights to adjust the regression models of interest. IPW was performed using Stata 14. Due to the potential for extreme weighted values adversely influencing subsequent analyses, larger weights were trimmed to 10.

Table S1. *Total, direct and indirect effects of parental alcohol use on young adult alcohol use (n=3,785)*

|  |  | Maternal alcohol use | | | | Partner alcohol use | | | | |
| --- | --- | --- | --- | --- | --- | --- | --- | --- | --- | --- |
|  | Moderate-risk | |  | High-risk |  | | Moderate-risk |  | High-risk |  |
|  | *b* (95% CI) | | *p* | *b* (95% CI) | *p* | | *b* (95% CI) | *p* | *b* (95% CI) | *p* |
| Total effect | 0.99 (.55, 1.43) | | <.001 | 1.74 (1.06, 2.46) | <.001 | | 1.10 (.70, 1.48) | <.001 | 1.02 (.18, 1.92) | .02 |
|  |  | |  |  |  | |  |  |  |  |
| Total indirect effect | 0.27 (.10, .46) | | <.01 | 0.49 (.18, .71) | <.01 | | 0.43 (.28, .61) | <.001 | 0.37 (.08, .69) | .02 |
|  |  | |  |  |  | |  |  |  |  |
| Specific indirect effects |  | |  |  |  | |  |  |  |  |
| Parental monitor | 0.03 (-.01, .08) | | .23 | 0.07 (.01, .15) | .06 | | 0.05 (.01, .10) | .03 | 0.06 (-.01, .16) | .17 |
| Early alcohol initiation | 0.15 (.06, .28) | | <.01 | 0.22 (.08, .40) | <.01 | | 0.25 (.15, .38) | <.001 | 0.19 (.03, .39) | .03 |
| Parental monitor–peer deviance | 0.02 (-.01, .05) | | .22 | 0.05 (.00, .10) | .05 | | 0.03 (.01, .07) | .03 | 0.04 (-.01, .10) | .17 |
| Early alcohol use–peer deviance | 0.07 (.03, .12) | | <.01 | 0.10 (.04, .17) | <.01 | | 0.11 (.07, .16) | <.001 | 0.08 (.01, .17) | .04 |
|  |  | |  |  |  | |  |  |  |  |
| Direct effect | 0.73 (.32, 1.15) | | .001 | 1.31 (.60, 2.03) | <.001 | | 0.66 (.26, 1.03) | <.001 | 0.65 (-.21, 1.54) | .15 |

Note: Unstandardized coefficients and 95% confidence intervals are presented; models adjusted for gender, maternal age at delivery, maternal smoking in pregnancy, maternal education, maternal depression in pregnancy, socioeconomic position, housing tenure, family income, and parity. Standard errors (and bias-corrected CIs) for indirect effects were calculated using bootstrapping with 5,000 replications.

Table S2. *Total, direct and indirect effects of parental alcohol use on young adult alcohol use (complete case sample - n=1,994)*

|  |  | Maternal alcohol use | | | | Partner alcohol use | | | | |
| --- | --- | --- | --- | --- | --- | --- | --- | --- | --- | --- |
|  | Moderate-risk | |  | High-risk |  | | Moderate-risk |  | High-risk |  |
|  | *b* (95% CI) | | *p* | *b* (95% CI) | *p* | | *b* (95% CI) | *p* | *b* (95% CI) | *p* |
| Total effect | 0.94 (.48, 1.39) | | <.001 | 1.61 (.94, 2.27) | <.001 | | 1.23 (.81, 1.67) | <.001 | 1.03 (.25, 1.81) | .01 |
|  |  | |  |  |  | |  |  |  |  |
| Total indirect effect | 0.20 (-.03, .43) | | .08 | 0.51 (.14, .88) | <.01 | | 0.43 (.22, .65) | <.001 | 0.46 (.08, .85) | .02 |
|  |  | |  |  |  | |  |  |  |  |
| Specific indirect effects |  | |  |  |  | |  |  |  |  |
| Parental monitoring | 0.03 (-.03, .09) | | .28 | 0.02 (-.07, .11) | .64 | | 0.05 (-.01, .10) | .10 | 0.04 (-.05, .14) | .35 |
| Early alcohol initiation | 0.11 (-.03, .25) | | .13 | 0.34 (.12, .56) | <.01 | | 0.26 (.12, .40) | <.001 | 0.28 (.04, .53) | .02 |
| Parental monitoring–peer deviance | 0.02 (-.02, .06) | | .27 | 0.01 (-.05, .07) | .64 | | 0.03 (.00, .06) | .09 | 0.03 (-.03, .09) | .35 |
| Early alcohol use–peer deviance | 0.04 (-.01, .09) | | .12 | 0.13 (.05, .21) | <.01 | | 0.10 (.05, .15) | <.001 | 0.11 (.01, .20) | .02 |
|  |  | |  |  |  | |  |  |  |  |
| Direct effect | 0.73 (.30, 1.17) | | .001 | 1.10 (.44, 1.76) | .001 | | 0.80 (.39, 1.21) | <.001 | 0.57 (-.24, 1.38) | .17 |

Note: Unstandardized coefficients and 95% confidence intervals are presented; models adjusted for gender, maternal age at delivery, maternal smoking in pregnancy, maternal education, maternal depression in pregnancy, socioeconomic position, housing tenure, family income, and parity

*Secondary analyses*

About 20% of the association between maternal alcohol use and hazardous alcohol use in young adulthood was accounted for by the hypothesized mediators. The majority of this association was accounted for by early alcohol initiation (moderate alcohol use: *b*=.037, 95% CI=.01, .07, *p*=.01; high-risk alcohol use: *b*=.061, 95% CI=.02, .11, *p*<.01). Between 34% and 47% of the association between partner alcohol use and hazardous use in young adulthood was accounted for by the same mediators. Again, the majority of this association was accounted for by early alcohol initiation (moderate alcohol use: *b*=.068, 95% CI=.04, .10, *p*<.001; high-risk alcohol use: *b*=.058, 95% CI=.01, .11, *p=*.02). Results focusing on the hazardous alcohol use outcome are presented in Supplementary Material (Table S3).

**Secondary Analyses: Hazardous alcohol use**

Table S3 shows strong evidence of a total indirect effect from maternal alcohol use to young adult alcohol use through parental monitoring, early alcohol use, and peer deviance (overall indirect effect from maternal moderate alcohol use: *b*=.29, 95% CI=.17, .41, *p*<.001; and maternal high-risk alcohol use: *b*=.48, 95% CI=.29, .67, *p*<.001). When examining the specific indirect effects for maternal alcohol use, the majority of the indirect was accounted for through early alcohol use (moderate alcohol use: *b*=.04, 95% CI=.01, .07, *p*=.01; high-risk alcohol use: *b*=.06, 95% CI=.02, .11, *p*=.01), and early alcohol use combined with peer deviance (moderate alcohol use: *b*=.01, 95% CI=.00, .02, *p*<.01; high-risk alcohol use: *b*=.02, 95% CI=.01, .03, *p*<.01). There was further strong evidence of a remaining direct effect from maternal alcohol use to harmful drinking in young adulthood after accounting for the mediators (moderate alcohol use: *b*=.23, 95% CI=.11, .35, *p*<.001; high-risk alcohol use: *b*=.38, 95% CI=.19, .56, *p*<.001).

In general, we found a similar pattern of findings when examining partner alcohol use. There was weak evidence to suggest that parental monitoring accounted for some of the association between partner alcohol use and young adult alcohol use (moderate alcohol use: *b*=.01, 95% CI=.00, .03, *p*=.03; high-risk alcohol use: *b*=.02, 95% CI=.00, .04, *p*=.07), and through parental monitoring and peer deviance (moderate alcohol use: *b*=.01, 95% CI=.00, .01, *p*=.02; high-risk alcohol use: *b*=.01, 95% CI=.00, .02, *p*=.05). There was further weak evidence of a remaining direct effect (moderate alcohol use: *b*=.12, 95% CI=.01, .23, *p*<.03; high-risk alcohol use: *b*=.21, 95% CI=-.01, .42, *p*=.06).

Table S3. *Total, direct and indirect effects of parental alcohol use on young adult hazardous alcohol use (n=3,785)*

|  |  | Maternal alcohol use | | | | Partner alcohol use | | | |
| --- | --- | --- | --- | --- | --- | --- | --- | --- | --- |
|  | Moderate-risk | |  | High-risk |  | Moderate-risk |  | High-risk |  |
|  | *b* (95% CI) | | *p* | *b* (95% CI) | *p* | *b* (95% CI) | *p* | *b* (95% CI) | *p* |
| Total effect | 0.29 (.17, .41) | | <.001 | 0.48 (.29, .67) | <.001 | 0.24 (.12, .35) | <.001 | 0.31 (.10, .53) | <.01 |
|  |  | |  |  |  |  |  |  |  |
| Total indirect effect | 0.06 (.02, .11) | | .01 | 0.10 (.04, .17) | <.01 | 0.11 (.07, .16) | <.001 | 0.11 (.03, .18) | <.01 |
|  |  | |  |  |  |  |  |  |  |
| Specific indirect effects |  | |  |  |  |  |  |  |  |
| Parental monitor | 0.01 (-.00, .02) | | .18 | 0.02 (-.00, .03) | .12 | 0.01 (.00, .03) | .03 | 0.02 (.00, .04) | .07 |
| Early alcohol use | 0.04 (.01, .07) | | .01 | 0.06 (.02, .11) | .01 | 0.07 (.04, .10) | <.001 | 0.06 (.01, .11) | .02 |
| Parental monitor–peer deviance | 0.01 (-.00, .01) | | .18 | 0.01 (-.00, .02) | .11 | 0.01 (.00, .01) | .02 | 0.01 (.00, .02) | .05 |
| Early alcohol use–peer deviance | 0.01 (.00, .02) | | <.01 | 0.02 (.01, .03) | <.01 | 0.02 (.01, .03) | <.001 | 0.02 (.00, .03) | .02 |
|  |  | |  |  |  |  |  |  |  |
| Direct effect | 0.23 (.11, .35) | | <.001 | 0.38 (.19, .56) | <.001 | 0.12 (.01, .23) | .03 | 0.21 (-.01, .42) | .06 |

Note: Unstandardized coefficients and 95% confidence intervals are presented; models adjusted for gender, maternal age at delivery, maternal smoking in pregnancy, maternal education, maternal depression in pregnancy, socioeconomic position, housing tenure, family income, and parity
